# Relationships of Disability with Age Among Adults Aged 50 to 85: Evidence from the United States, England and Continental Europe

**DOI:** 10.1371/journal.pone.0071893

**Published:** 2013-08-14

**Authors:** Morten Wahrendorf, Jan D. Reinhardt, Johannes Siegrist

**Affiliations:** 1 International Centre for Life Course Studies in Society and Health (ICLS), Research Department of Epidemiology and Public Health, University College London, London, United Kingdom; 2 Swiss Paraplegic Research, Nottwil, Switzerland; 3 University of Lucerne, Department of Health Sciences & Health Policy, Lucerne, Switzerland; 4 Senior Professorship on Work Stress Research, Faculty of Medicine, University of Duesseldorf, Duesseldorf, Germany; Old Dominion University, United States of America

## Abstract

**Objectives:**

To extend existing research on the US health disadvantage relative to Europe by studying the relationships of disability with age from midlife to old age in the US and four European regions (England/Northern and Western Europe/Southern Europe/Eastern Europe) including their wealth-related differences, using a flexible statistical approach to model the age-functions.

**Methods:**

We used data from three studies on aging, with nationally representative samples of adults aged 50 to 85 from 15 countries (N = 48225): the US-American Health and Retirement Study (HRS), the English Longitudinal Study of Ageing (ELSA) and the Survey of Health, Ageing and Retirement in Europe (SHARE). Outcomes were mobility limitations and limitations in instrumental activities of daily living. We applied fractional polynomials of age to determine best fitting functional forms for age on disability in each region, while controlling for socio-demographic characteristics and important risk factors (hypertension, diabetes, obesity, smoking, physical inactivity).

**Results:**

Findings showed high levels of disability in the US with small age-related changes between 50 and 85. Levels of disability were generally lower in Eastern Europe, followed by England and Southern Europe and lowest in Northern and Western Europe. In these latter countries age-related increases of disability, though, were steeper than in the US, especially in Eastern and Southern Europe. For all countries and at all ages, disability levels were higher among adults with low wealth compared to those with high wealth, with largest wealth-related differences among those in early old age in the USA.

**Conclusions:**

This paper illustrates considerable variations of disability and its relationship with age. It supports the hypothesis that less developed social policies and more pronounced socioeconomic inequalities are related to higher levels of disability and an earlier onset of disability.

## Introduction

The ‘compression of morbidity’ hypothesis claims that the age of onset of major chronic diseases and of disability has been postponed in recent years, with a sharpening increase in later life and most of the morbidity in life squeezed into the years before death [1]. It is argued that this is mainly due to increased preventive efforts and advances in detecting and treating chronic disorders. While the hypothesis received limited support with regard to chronic diseases until now, there is support for a compression of disability [2], [3]. Yet, evidence is mainly restricted to adults with healthy life styles and most studies come from the US [4], [5].

A growing body of research compared health between the US, England and continental European countries, demonstrating important health disadvantages of older adults in the US compared to their European counterparts [6], [7], [8], [9], [10]. These differences exist for distinct chronic diseases (for example diabetes, hypertension and stroke) [6], [7], [8] and for disability and functional limitations [6], [9]. Other studies report variations in health across European countries or regions, with poorer health in Southern and Eastern Europe compared to Northern and Western Europe [10], [11], [12]. However, while these studies compare the overall health levels of broad age groups, less information so far is available on age-related increases of poor health, and in particular on the age-function of disability.

At the same time, there is robust evidence of socioeconomic differences in level and age of onset of disability, leaving those in lower socioeconomic positions at higher risk [13], [14], [15], [16], [17]. Again, a striking finding of comparative studies reveals more pronounced differences in the US and England compared to other European countries [6], [7]. The challenges of demographic aging [18], [19] and the associated financial burden of disability require to elucidate the relationship between age and disability across different countries and regions, including socio-economic differences.

While cohort studies are considered the gold standard of analysing age-related changes in human functioning and disability onset at the individual level, cross-sectional comparative studies of older populations provide valuable supplementary information. Despite the fact that age effects and cohort effects cannot be separated, cross-sectional data from comparative studies inform about differences between countries and about the different steepness of their increase with age. With the recent availability of comparable studies on aging in different parts of the world [20], [21], [22] new opportunities exist to explore these differences and to discuss potential scientific and policy-related implications.

Along these lines we set out, first, to analyse the relationships of disability with age in the US, England and continental Europe based on comparative data from three studies, HRS, ELSA and SHARE (see Methods). More specifically, we investigate the age-disability relationship across five major regions (US in HRS, England in ELSA, Northern and Western Europe, Southern, and Eastern Europe in SHARE) by applying a statistical approach (fractional polynomials for age) that allows for flexible modulation of the age function for each region (see Methods for details). This approach does neither assume a linear association between age and disability, nor does it loose information by classifying people into age-groups. Rather, it provides flexible estimates of the function of age for each region. As a second aim, we examine socio-economic differences by studying age-disability relationships according to wealth, again separately for the above regions.

## Methods

### Data Sources

We used data from three international studies on aging, with information collected 2006 in 15 countries, the US-American Health and Retirement Study (HRS) [21], the ‘English Longitudinal Study of Ageing’ (ELSA) [23] and the ‘Survey of Health, Ageing and Retirement in Europe’ (SHARE) [20]. The three studies were developed in close coordination, with a focus on harmonization of research methods and study designs to allow for cross-national comparisons. All studies consist of nationally representative samples of individuals aged 50 and older, and they cover a variety of sociological, economic and health-related topics. Samples are based on probability household samples (either drawn as simple random selection or multistage random selection) and respondents are interviewed using Computer Assisted Personal Interviews (CAPI) (see references above for details). While HRS started 1992, ELSA began 2002, and the first wave of SHARE was 2004 (in 11 countries). All three studies have on-going waves of data collection in two-year intervals, with new cohorts (so called “refreshers”) being added subsequently to maintain population representation. In 2006 two new countries joined SHARE (Czech Republic and Poland). Household response rates at study onset were 80% for HRS, 70% for ELSA and 61% for the total SHARE sample in 2004 ranging from 39% in Switzerland to 81% in France. Attrition rates between 2002 and 2006 were 27% in case of ELSA (between wave 1 and wave 3) and 5% for the same time window in HRS (between wave 6 and wave 8) [24]. In case of SHARE 28% were lost between wave 1 and wave 2 [25]. By combining 2006 cross-sectional data from the three surveys in this analysis, highest possible number of countries (and participants) was achieved. This serves our aim to study the age-disability relationship in different countries ranging from the US, England, Northern Europe (Sweden and Denmark), Western Europe (Germany, the Netherlands, Belgium, France, Switzerland and Austria), Southern Europe (Italy, Spain and Greece) and Eastern Europe (the Czech Republic and Poland). For the analysis, we reduced our sample to respondents aged 50 to 85 years and excluded individuals with missing data on measures of disability and the remaining covariates, resulting in a total sample of 48,225 men and women (SHARE = 30,395, ELSA = 6,141, HRS = 11,689).

HRS was approved by the institutional review board from the University of Michigan Health Services. SHARE was approved by the institutional review board at University of Mannheim, Germany. Ethical approval for ELSA was obtained from the Multi-Centre Research Ethics Committees in the United Kingdom.

### Measures

#### Disability

We used two measures of disability. Both measures are based on identical measurement procedures in all three studies (including identical wording and response categories in the questionnaires). They were used in previous cross-national studies [6] and were shown to be well comparable between the three studies [26]. The first measure counts the number of reported limitations in mobility (‘Mobility limitations’), based on a list of 10 items [27]. These limitations include difficulties in mobility, arm functions and fine-tuned motor function. The second measure indicates the number of limitations in performing instrumental activities of daily living (‘IADL limitations’), based on 6 activities [28]. Compared to mobility limitations, IADL limitations are considered more severe as they impair the performance of essential activities of an independent life. In case of mobility the sum score ranges from 0 to 10, and in case of IADL limitations from 0 to 6, with higher values indicating higher levels of disability. All items are shown in the supporting information (Table S1).

#### Additional measures

In addition to age and sex, we included two sets of additional variables.

First these were retirement status, partnership situation, education and wealth. Retirement status is based on the self-reported employment situation. A binary indicator measures whether the respondent lived in a partnership (without considering the marital status). To measure education and wealth we applied the same procedures as other comparative studies [6], [7], [29]. In SHARE and ELSA education was measured according to the International Standard Classification of Educational Degrees (ISCED-97) that was regrouped into ‘low education’ (pre-primary, primary or lower secondary education), ‘medium education’ (secondary or post-secondary education), and ‘high education’ (first and second stage of tertiary education). In the HRS study corresponding levels were obtained based on years of education (‘low education’: 0–11 years, ‘medium education’: 12–15 years, ‘high education’: 16 and more years). Our measure of wealth refers to household total net worth, which we adjusted for household size in accordance to the OECD equivalent-scale, and thereafter categorised into country-specific tertiles (low, medium, high). In addition to financial wealth (savings, net stock value, mutual funds and bonds), it also includes housing wealth (value of primary residence, other real estates and own business share and cars). Thus, it includes accumulated savings and not only direct income, which may be more appropriate for older populations and less confounded by country-specific policies (e.g. pension policies).

As a second set of additional variables we included the following important risk factors, again following measurement procedures of previous studies [6], [7], [29]. First, we used respondents’ body mass index (BMI, calculated as weight in kilograms divided by the square of height in meters) and created an indicator of obesity (BMI 30 or higher). Information on weight and height was either self-reported (HRS, SHARE) or measured (ELSA) and measures that were not regarded as reliable by the interviewer were categorized as ‘not reliable’. Second, a binary indicator of current smoking status (yes/no) was included. In addition, we included an indicator of physical inactivity, defined as never or almost never engaging in moderate and vigorous physical activity. Finally, we considered histories of two major chronic diseases (diabetes and hypertension), measured by self-reported doctor diagnoses. A description of all variables and the sample is given for each study in Table 1.

**Table 1:** Sample description: Frequencies or mean scores (Standard Deviation, SD) in HRS, ELSA and SHARE.

Variables	Categories or range	HRSN = 11689	ELSAN = 6141	SHAREN = 30395
*Mean mobility limitations*	0–10	2.39 (2.60)	1.95 (2.52)	1.46 (2.18)
*Mean IADL limitations*	0–6	0.32 (0.90)	0.23 (0.73)	0.20 (0.75)
*Mean Age*	50–85	66.17 (8.75)	66.46 (8.60)	64.59 (9.16)
*Sex %*	Male	42.28	46.41	45.53%
	Female	57.72	53.59	54.47%
*Education %*	Low	17.84	42.88	48.50
	Medium	57.00	27.63	33.13
	High	25.16	29.49	18.36
*Wealth %*	Low	33.36	31.83	33.37
	Medium	33.37	33.63	33.34
	High	33.27	34.54	33.29
*Partnership %*	Yes	69.03	70.10	76.61
	No	30.97	29.90	23.39
*Retired %*	Yes	57.15	56.57	50.99
	No	42.85	43.43	49.01
*Obesity %*	Yes	30.05	28.48	18.33
	No	68.69	67.06	80.09
	not reliable	1.26	4.46	1.58
*Diabetes %*	Yes	18.63	9.92	10.42
	No	81.37	90.08	89.58
*Hypertension %*	Yes	54.28	34.82	34.74
	No	45.72	65.18	65.26
*Current smoking %*	Yes	14.00	13.84	20.74
	No	86.00	86.16	79.26
*Physical inactivity %*	Yes	15.98	15.70	10.21
	No	84.02	84.30	89.79

### Statistical Analyses

**Figure 1:**
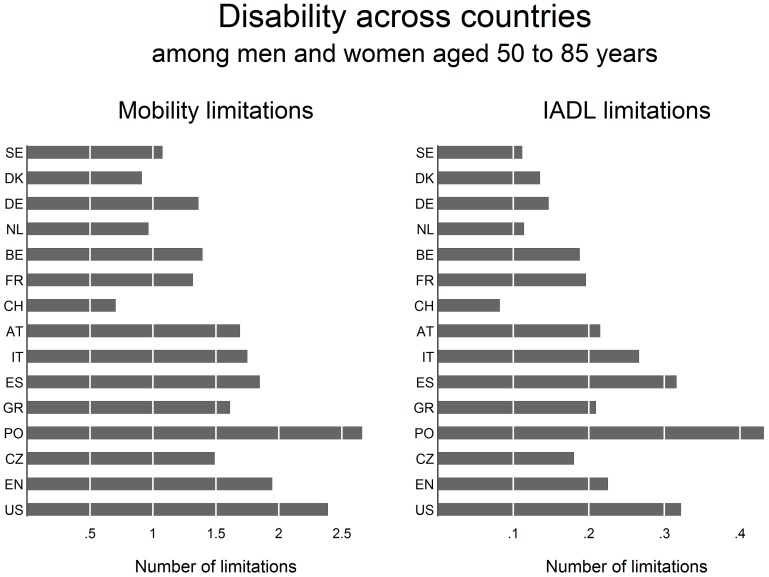
Mean numbers of disability (unadjusted) across countries. Note. (Sweden, SE; Denmark, DK; Germany, DE; Netherlands, NL; Belgium, BE; France, FR, Switzerland, CH; Austria, AT; Italy, IT; Spain, ES; Greece, GR; Poland, PO; Czech Republic, CZ; England, EN; United States, US).

Following descriptive analyses, we compared average numbers of disability limitations for each country (Figure 1). Subsequent analyses further divided SHARE countries into three subgroups, the group with relatively low levels (Northern and Western Europe), and two groups with relatively high levels of disability, but different political histories (Southern Europe “SHARE south” and Eastern Europe “SHARE east”).

**Figure 2:**
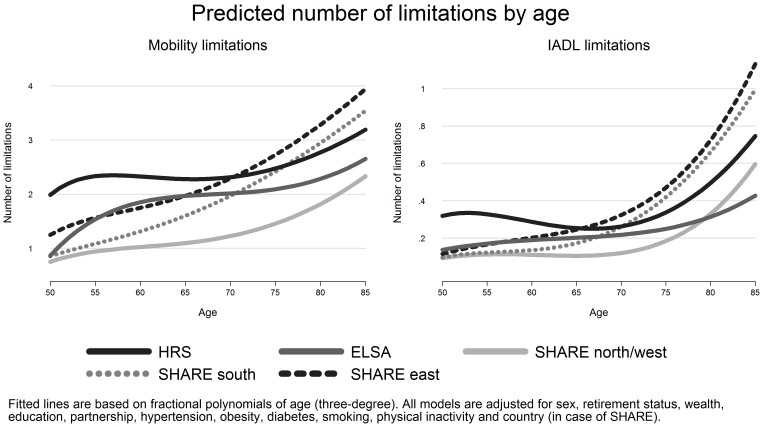
Predicted number of disability limitations by age (adjusted). Note. All models are adjusted for sex, education, wealth, retirement status, partnership, hypertension, diabetes, obesity, smoking, physical inactivity and country (in case of SHARE). “SHARE north/west” includes Sweden, Denmark, Germany, Austria, Netherlands, Belgium, France and Switzerland; “SHARE south” includes Italy, Spain and Greece, “SHARE east” includes Poland and Czech Republic.

**Figure 3:**
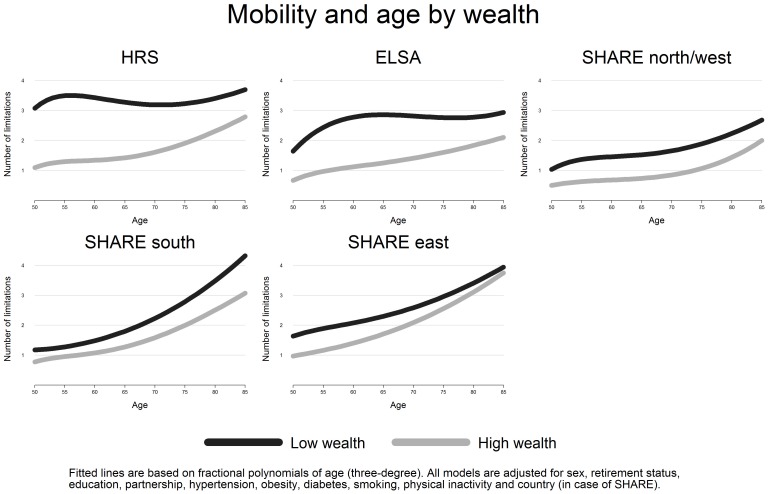
Predicted number of mobility limitations by age and wealth (adjusted). Note. All models are adjusted for sex, education, retirement status, partnership, hypertension, diabetes, obesity, smoking, physical inactivity and country (in case of SHARE). “SHARE north/west” includes Sweden, Denmark, Germany, Austria, Netherlands, Belgium, France and Switzerland; “SHARE south” includes Italy, Spain and Greece, “SHARE east” includes Poland and Czech Republic.

**Figure 4:**
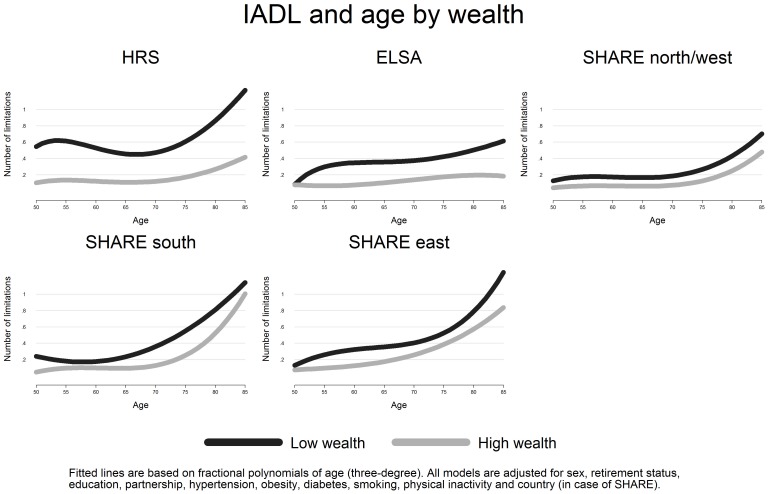
Predicted number of IADL limitations by age and wealth (adjusted). Note. All models are adjusted for sex, education, retirement status, partnership, hypertension, diabetes, obesity, smoking, physical inactivity and country (in case of SHARE). “SHARE north/west” includes Sweden, Denmark, Germany, Austria, Netherlands, Belgium, France and Switzerland; “SHARE south” includes Italy, Spain and Greece, “SHARE east” includes Poland and Czech Republic.

Next, we estimated ordinary least squares (OLS) regression models to analyse the age- disability relationship for each country group with number of disability limitations as outcomes. In these analyses we used fractional polynomial (FP) transformations of age [30], [31], [32] and the “fracpoly” procedure in STATA. This serves our aim to allow for a non-linear modelling of age. In contrast to standard polynomials (for example age square), power terms of fractional polynomials are not limited to positive integers only, but may include negative and fractional powers as well. Therefore, a wider set of possible functional forms of age is provided, offering more flexibility than standard polynomials. In addition, the use of FPs has several advantages compared to categorizing age into age-groups [30]. For example, it avoids an inflated number of parameters. Or, instead of treating all cases within an age group as being equal, it accounts for possible variations of disability within age groups. Similarly, while the use of age-categories allows to study trends between the chosen age-groups only (often defined arbitrarily), FPs do respect the possibility that cut-off points (and turning points) may vary between subpopulations [31]. In comparison to further approaches modelling non-linear functions (e.g spline-models) FP are also easier to implement, and simulations studies indicate favourable performance [33]. An important aspect of FPs and the “fracpoly” procedure relates to the selection of power terms. Instead of being defined by the researcher (as in the case of standard polynomials), power terms are selected by an automated process (for each country group in our case). More specifically, a large number of regression models are estimated using different power terms for age (and combination of power terms) to identify the model that best describes disability as a function of age. To compare these models deviance tests are used and power terms are selected from the default setting (−2, −1, −0.5, 0, 0.5, 1, 2, 3), where 0 denotes the log transformation. In our analyses, we included three fractional polynomials for age (third degree FP) allowing for two possible turning points and comparing 164 models to select best fitting power terms. Models were adjusted for socio-demographic characteristics and important risk factors (hypertension, diabetes, obesity, smoking, physical inactivity). The resulting power terms are presented in the Results section (Table 2), and we contrast the models against a model without age (to test for significance of age) and against a model that includes linear age (to test for non-linearity), using deviance differences. Besides, we formally analyzed interactions between country-groups and age, by combining the data, estimating common fractional polynomials of age and then testing interactions between age and country groups using likelihood ratio tests [34]. Since estimated coefficients for the fractional polynomials are difficult to interpret [32], we display resulting curves of the age-disability relationship in Figure 2 [35]. Finally, we studied associations between wealth and the two disability indicators, presenting two models in Table 3. Model 1 included wealth, gender and the fractional polynomials for age. Model 2 was additionally adjusted for all confounders mentioned above. Finally, Figure 3 and 4 present the age-disability relationship for highest and lowest wealth levels for each country group. All findings remained unchanged when multivariate Poisson regression models were used. Calculations and graphs were produced with STATA 12.

**Table 2:** Adjusted fractional polynomial models for the relationship between age (3-degree) and disability: Best powers for age and tests of significance.

	HRS	ELSA	SHARE		
	US	England	north/west	south	east
	N = 11689	N = 6135	N = 17835	N = 7627	N = 4933
**Mobility limitations**					
Best powers for age	−2, −2, 1	1, 1, 1	0.5, 0.5, 0.5	−1, −1, −1	−2, −1, −1
Deviance difference to model without age (p-value)	81.054 (0.000)	53.568 (0.000)	436.015 (0.000)	549.399 (0.000)	252.330 (0.000)
Deviance dif.to model with linear age (p-value)	38.895 (0.000)	10.754 (0.057)	72.606 (0.000)	26.828 (0.000)	18.774 (0.002)
**IADL limitations**					
Best powers for age	−2, −0.5, 3	3, 3, 3	3, 3, 3	0.5, 0.5, 1	3, 3, 3
Deviance difference to model without age (p-value)	147.586 (0.000)	30.519 (0.000)	390.101 (0.000)	350.472 (0.000)	211.117 (0.000)
Deviance dif.to model with linear age (p-value)	107.155 (0.000)	6.698 (0.245)	190.091 (0.000)	96.362 (0.000)	50.940 (0.000)

Note.

All models are adjusted for sex, education, wealth, retirement status, partnership, hypertension, diabetes, obesity, smoking, physical inactivity and country (in case of SHARE).

“SHARE north/west” includes Sweden, Denmark, Germany, Austria, Netherlands, Belgium, France and Switzerland; “SHARE south” includes Italy, Spain and Greece, “SHARE central” includes Poland and Czech Republic.

**Table 3:** Associations between wealth and disability: results of regression models (unstandardized regression coefficients (b), significant levels and standard errors (SE)).

		HRS		ELSA		SHARE					
		US		England		north/west		south		east	
		N = 11689		N = 6135		N = 17835		N = 7627		N = 4933	
		b	(SE)	b	(SE)	b	(SE)	b	(SE)	b	(SE)
**Mobility limitations**											
Model 1	Low wealth	1.791***	0.056	1.371***	0.077	0.708***	0.034	0.529***	0.059	0.448***	0.077
	Medium wealth	0.616***	0.049	0.547***	0.066	0.277***	0.030	0.256***	0.056	0.263***	0.074
	High wealth	–		–		–		–		–	
Model 2	Low wealth	0.883***	0.057	0.578***	0.072	0.393***	0.032	0.246***	0.056	0.229**	0.071
	Medium wealth	0.291***	0.046	0.233***	0.063	0.150***	0.028	0.105*	0.052	0.136*	0.068
	High wealth	–		–		–		–		–	
**IADL limitations**											
Model 1	Low wealth	0.446***	0.021	0.255***	0.024	0.107***	0.011	0.158***	0.023	0.133***	0.029
	Medium wealth	0.100***	0.016	0.066***	0.018	0.033***	0.009	0.066**	0.021	0.056*	0.026
	High wealth	–		–		–		–		–	
Model 2	Low wealth	0.219***	0.021	0.094***	0.022	0.042***	0.011	0.086***	0.022	0.067*	0.028
	Medium wealth	0.033*	0.015	0.003	0.017	0.009	0.009	0.030	0.020	0.028	0.025
	High wealth	–		–		–		–		–	

Note. * p<0.05; ** p<0.01; *** p<0.001.

Models 1 is adjusted for gender, age (3-degree FP), and country affiliation (in case of SHARE). Model 2 additionally includes education, retirement status, partnership, hypertension, diabetes, obesity, smoking and physical inactivity. “SHARE north/west” includes Sweden, Denmark, Germany, Austria, Netherlands, Belgium, France and Switzerland; “SHARE south” includes Italy, Spain and Greece, “SHARE central” includes Poland and Czech Republic.

## Results

### Sample Description

Table 1 presents the samples for England (using ELSA), the US (using HRS) and continental Europe (using SHARE). The largest sample is SHARE (N = 30395), followed by HRS (N = 11689) and ELSA (N = 6141). By and large, study samples are similar with regard to socio-demographic characteristics, where frequencies of women are slightly higher compared to men. Mean age ranges between 64 and 66 years, and a large majority of respondents live in a partnership. Retirement rates are somewhat higher in case of England and the US compared to continental Europe. Rates of obesity, diabetes, hypertension and physical inactivity were highest in the US (confirming previous findings [6], [7], [8]).

Turning to both measures of disability, average numbers are highest in the US, followed by England and continental Europe – again a finding that is in line with previous studies [6]. For example, US respondents report on average more than two mobility limitations, whereas values are below two in the remaining studies. In case of IADL limitations, mean numbers were generally lower, indicating lower rates of severe disability limitations in the samples. Yet again, we observe a higher level in HRS, followed by ELSA and SHARE. To further explore whether these differences hold true for each SHARE country, Figure 1 presents the average number of disability limitations for each country.

At first glance, levels of disability are again generally lower for SHARE countries compared to ELSA and HRS (particularly for Northern and Western Europe). However, overall levels are rather high for Eastern and Southern Europe, with Poland exceeding even the levels observed in England and the US. Yet, it is possible that high levels of disability result from a particularly high prevalence of disability among the oldest old (as claimed by the compression of morbidity hypothesis). To analyse this problem we estimate the age-function of disability and account for the differences within SHARE countries by regrouping SHARE into three groups, “SHARE north/west” (Northern and Western Europe: Sweden, Denmark, Germany, Austria, Netherlands, Belgium, France and Switzerland), “SHARE south” (Southern Europe: Italy, Spain, Greece) and “SHARE east” (Eastern Europe: Poland and Czech Republic).

### Age-disability Relationship

Before turning to the predicted curves (figure 2), we briefly describe Table 2. This table lists best fitting power terms of the three FPs of age (adjusted for all confounders), together with the results testing their significance (a) against a model without age, and (b) against a model where age is included as linear term. First, we observe that powers terms vary between countries and, as expected, that age is significantly associated with both disability measures in all country groups. Additionally, we find clear support for non-linear relationships. In one case only (IADL limitations for the English sample), the model fit of the FP model does not increase significantly compared to a linear model, thus suggesting an almost linear increase of IADL limitations with age in England. Additional analyses combining all data and testing interactions between age and country groups confirmed that age-functions were significantly different between country groups (for both measures of disability).

The following three observations deserve attention in figure 2. First, levels of disability are again highest in the US (dark solid line), with two mobility limitations manifested already before the age of 60. Second, while slopes are similar for the US, England and “SHARE north/west”, a steeper increase of limitations is evident in Eastern and Southern Europe (dashed and dotted lines). In these latter countries, levels of disability exceed those observed in the US among people aged 75 to 85, in particular in case of the two Eastern European countries (Poland and Czech Republic). As a third observation, slopes are more pronounced in case of IADL limitations than in case of mobility limitations, and the onset of remarkable limitations occurs at a later stage of the life course, compared to mobility limitations. In supplementary analyses, we tested for interactions between age and sex within each country group. While main effects revealed significant higher levels of disability for women throughout all ages, slopes were similar to the ones presented in figure 2. Shapes were only slightly different within “SHARE south” (somewhat later onset for men) and “SHARE north/west” (slightly steeper increase in later ages for women), but no differences were found in case of the US, England, and the eastern countries (for both measures of disability).

### Wealth Differences

Table 3 lists estimated coefficients for the two lower wealth groups versus the highest wealth group. In model 1 the coefficients are adjusted for gender, age and country affiliation (in case of SHARE groups only). In all cases, levels of disability are significantly higher among people with low wealth compared to those at the top of the wealth distribution. Yet, these wealth-differences are largest in the US, followed by England and the SHARE groups. For instance, US Americans in the lowest wealth group report on average 1.8 more mobility limitations compared to people with high wealth, while differences range between 0.4 and 0.7 in the SHARE groups. Importantly, Model 2 shows that the included confounders (education, retirement status, partnership, hypertension, obesity, diabetes, smoking, physical inactivity) can only partly account for these wealth–differences, i.e. for differences in IADL limitations between middle and high wealth groups. In further analyses, we combined the data and tested interactions between country groups and wealth. Results (not shown in detail) confirm that wealth-differences were significantly larger in the US and in England compared to the three SHARE groups. In a final step, we present the age-disability relationship for the highest and the lowest wealth group, once more for each country group (Figure 3 and 4).

Again, wealth differences are striking in the US, where the low wealth group is characterized by three mobility limitations already at age 50 (Figure 3, dark line), and where an age-related increase is less pronounced compared to the higher wealth group (. A similar pattern (though at a lower mean level) is observed in the English sample: High wealth groups exhibit clearly lower levels of mobility limitations compared to people reporting low wealth, specifically in the age range 55 and 65.

## Discussion

Four major findings result from our analyses. First, we documented substantial differences in average numbers of disability limitations in older age between the five regions under study. Levels of disability were generally higher among older adults in the US, followed by Eastern Europe, England, Southern Europe, and lowest in Northern and Western Europe (for mobility limitations and IADL limitations). Second, by modeling the age-function of disability for each region using fractional polynomials of age, we discovered meaningful differences of the age-disability relationship for both indicators. In contrast to mobility limitations, the onset of remarkable amounts of IADL limitations generally occurred at a later stage of the life course, and significant increases are evident at advanced age only (70 years and older). With regard to differences between regions, age-related increases of disability were particularly steep in Eastern and Southern Europe where levels of disability, at least among people aged 75 to 85, exceeded those observed in the US which in turn had highest levels of disability at early old ages. Our third main finding was an inverse association of level of disability and wealth, which was most pronounced in the US. Fourth, it became apparent that in countries with larger wealth-differences in disability (the US and England), the highest wealth groups experience more disability-free years as an age-related increase is observed at advanced age only.

These results are in line with previous findings [6], [7], [8], [9], but they add two important new elements. First, by including countries from Eastern Europe we analyzed the burden of disability in countries which faced major socio-economic and/or political challenges during an extended period of time before data collection, in contrast to economically and politically more consolidated countries. Second, we analyzed non-linear age-disability relationships for each region using fractional polynomials of age. This prevented us from categorizing respondents into age groups (with its loss of information) and allowed for a flexible modulation of the age-function for each subgroup. In doing so, we demonstrated that age-related increases in number of disability limitations differ between the five regions (steepest increases in the oldest age groups in Eastern and Southern Europe). Furthermore, this allowed us to demonstrate a later onset of mobility limitations among highest wealth groups in the US and England, compared to the remaining countries. Apart from the financial and socio-political implications this finding contradicts the compression of morbidity hypothesis, at least for older adults in lower socio-economic positions in the US.

How can we explain these differences in levels of disability between the countries under study, as well as the different shapes of disability throughout older ages? It seems unlikely that artefactual explanations can account for these findings (for example differences in reporting style and diagnosing or selective survival). Rather, we may consider at least three types of explanations.

First, albeit we adjusted for important confounders, the results may be due to different composition of the populations, including country-specific prevalence of additional health risk behaviors. For instance, in the US the group of immigrants and ethnic minorities is larger compared to Europe. Although research has documented a health advantage of this group of immigrants (in particular for Hispanic immigrants), length of stay in the US has been associated with a clear decline of health [36], and thus, this group in the long run may carry specific health risks. However, studies addressing this issue found only partial support in favor of this explanation [6], [7], [8], as differences in health persisted between the US and Europe after adjusting for ethnic group.

Different national policies provide a second explanation of the observed cross-country differences. Links between such policies and the prevalence of disability may be related to supportive or unsupportive health care systems, or they may be due to some intermediate policies affecting working conditions as well as the provision of social welfare and social security. For instance, in 2007 most European countries had universal health coverage, while in the US about 43 Million (17% of inhabitants younger than 65) were uninsured, with universal health programs (Medicare) only being offered to people aged 65 or older [37]. Furthermore, the US health system may pay less attention to prevention compared to Europe [38] and offer less compensation or benefit measures in case of disability [39]. The US belongs to the OECD countries with most stringent eligibility criteria for disability benefits and shortest payment duration [39]. To some extent, these policies may account for the high prevalence of disability in the US. Likewise, restricted welfare states and neoliberal policies have been associated with higher levels of morbidity and shorter life expectancy [40], [41], [42], [43], [44]. Further studies suggest that labor market policies play an important role [29], [45], [46]. These policies may indirectly be related to health, by promoting favourable working conditions (for example through active labor market policies or enacted regulations of occupational safety), or they may mitigate the health-adverse effects of poor working conditions (for example through employment protection policies). In case of Eastern Europe and the United States, these latter aspects seem to be less developed, thus inducing poorer working conditions and stronger health-adverse effects.

Third, our findings can emerge from more general societal circumstances. Critical circumstances include a weakness of the available safety nets [47], a tendency of social exclusion of older people [48], a widening of social inequalities in different life spheres (for example income and wealth) [49], or the differential exposure to the harshness of poverty [50]. All these factors vary between countries, where levels of inequality are higher in the US and England compared to continental Europe [51], and where poverty and social exclusion among older people are particularly high in Eastern and Southern Europe [48].

The proposed explanations clearly call for further research that needs to take into account the interconnectivity of social and economic, policy-related and health-related conditions. This analysis is even more challenging as levels of health and functioning among older people are additionally determined by conditions during earlier stages of the life course [52], in particular early life [53] and midlife [54], [55]. Hence, one challenge of future analyses is to focus explicitly on distinct policies and to address life course exposures when explaining later health. Similarly, while our analyses focused on differences between country groups, for some country groups we found support that associations between age and disability additionally vary by sex, and thus, future analyses may address this question in more detail.

Although our study profits from several strengths (comparative study design with identical measures, adjustments for important confounders, and flexible modulation of the age function for each country group by applying fractional polynomials), we have to consider several limitations. First, longitudinal data would have strengthened the findings, where individual trajectories can be studied instead of presenting age-specific levels of disability. However, comparative longitudinal information from all three studies was limited in our case, since data was either restricted to two waves in a majority of the cases (11 out of 15 countries), or respective data was not yet available in two-year intervals when this analysis was conducted (2 out of 15 countries). Second, survey participation was not very high in some countries (specifically in case of SHARE), and a selection bias may have affected our results to some extent, such that people in good health were more likely to participate. Yet, compared to European standards, response rates in the samples included were above average, and analyses comparing the SHARE sample to other prominent European surveys (e.g. the European Social Survey) confirmed that the sample represents the general population quite well [20]. In addition, as documented in a previous analysis [24], health-differences between the US and England could not be explained by different attrition rates. Third, although health differences (including biomarkers) between these countries were reported previously [7], [8], future research may extend our analysis focusing on more differentiated domains of disability, as defined by the International Classification of Functioning, Disability and Health (for example mental impairment) [56].

In conclusion, the results of this study demonstrate considerable variation of disability and, additionally, its relationship with age between countries and between socio-economic positions. Our results support the assumption that countries with less developed social policies and more pronounced social inequalities have higher levels of disabilities in older ages as well as an earlier onset of disability. The findings provide additional evidence in favor of targeted investments into healthy ageing.

## Supporting Information

Table S1 **Items used to create disability scales.** Note. ^1^ Because of a health problem, do you have difficulty doing any of the activities on this card? Exclude any difficulties that you expect to last less than three months. ^2^ Here are a few more everyday activities. Please tell me if you have any difficulty with these because of a physical, mental, emotional or memory problem. Again exclude any difficulties you expect to last less than three months.
(DOCX)
