# Supplementary material for: Relationships of Disability with Age Among Adults Aged 50 to 85: Evidence from the United States, England and Continental Europe
Source: PLoS One. 2013 Aug 14;8(8):e71893. doi: 10.1371/journal.pone.0071893 (PMC3743762; doi:10.1371/journal.pone.0071893)
Supplement: Table S1 — Items used to create disability scales. Note. 1 Because of a health problem, do you have difficulty doing any of the activities on this card? Exclude any difficulties that you expect to last less than three months. 2 Here are a few more everyday activities. Please tell me if you have any difficulty with these because of a physical, mental, emotional or memory problem. Again exclude any difficulties you expect to last less than three months. (DOCX) [file pone.0071893.s001.docx]

**Supporting information**

Table S1 - Items used to create disability scales

|  |
| --- |
| Mobility limitations ^1^ |
| 1. Walking 100 meters (100 yards) |
| 1. Sitting for about 2 hours |
| 1. Getting up from a chair after sitting for long periods |
| 1. Climbing several flights of stairs without resting |
| 1. Climbing one flights of stairs without resting |
| 1. Stooping, kneeling, or crouching |
| 1. Reaching or extending your arms above shoulder level |
| 1. Pulling or pushing large objects like a living room chair |
| 1. Lifting or carrying weights over 5 kilos (100 pounds) like a heavy bag of groceries |
| 1. Picking up a small coin from a table |
|  |
| Instrumental Activities of Daily Living (IADL limitations) ^2^ |
| 1. Using a map to figure out how to get around in a strange place |
| 1. Preparing a hot meal |
| 1. Shopping for groceries |
| 1. Making telephone calls |
| 1. Taking medications |
| 1. Managing money, such as paying bills and keeping track of expenses |
|  |

*Note.*

*^1^ Because of a health problem, do you have difficulty doing any of the activities on this card? Exclude any difficulties that you expect to last less than three months.*

*^2^ Here are a few more everyday activities. Please tell me if you have any difficulty with these because of a physical, mental, emotional or memory problem. Again exclude any difficulties you expect to last less than three months.*
